# Interictal Single-Voxel Proton Magnetic Resonance Spectroscopy of the Temporal Lobe in Dogs With Idiopathic Epilepsy

**DOI:** 10.3389/fvets.2020.00644

**Published:** 2020-09-24

**Authors:** Agnieszka Olszewska, Martin Jürgen Schmidt, Klaus Failing, Józef Nicpoń, Przemysław Podgórski, Marcin Adam Wrzosek

**Affiliations:** ^1^Department of Veterinary Clinical Science, Small Animal Clinic, Justus-Liebig-University Giessen, Giessen, Germany; ^2^Unit for Biomathematics and Data Processing, Faculty of Veterinary Medicine, Justus Liebig-University Giessen, Giessen, Germany; ^3^Department of Internal Diseases With a Clinic for Horses, Dogs and Cats, Faculty of Veterinary Medicine, Wrocław University of Environmental and Life Sciences, Wrocław, Poland; ^4^Center of Experimental Diagnostics and Innovative Biomedical Technologies, Faculty of Veterinary Medicine, Wrocław University of Environmental and Life Sciences, Wrocław, Poland; ^5^Department of General Radiology and Interventional Radiology and Neuroradiology, Wrocław Medical University, Wrocław, Poland

**Keywords:** dog, idiopathic epilepsy, MRI, MRS, seizures

## Abstract

Proton magnetic resonance spectroscopy (H1-MRS) could provide insight into the metabolic pathophysiology of the temporal lobe of canine brain after seizure. Currently, there is no evidence-based data available on MRS of temporal lobe in dogs with idiopathic epilepsy (IE). The aim of this prospective, cross-sectional study was to evaluate the interictal metabolic activity of the temporal lobe in IE dogs compared to a control group with the use of H1-MRS. Ten healthy dogs and 27 client-owned dogs with IE underwent 1.5-Tesla magnetic resonance imaging (MRI) and single-voxel H1-MRS. The MRS studies were acquired as spin echoes with a repetition time (TR) of 2,000 ms and an echo time (TE) of 144 ms. A cubic voxel (10 ×10 ×10 mm) was positioned bilaterally into the region of the left and right temporal lobe, including a middle part of the hippocampus and the amygdala. The N-acetylaspartate (NAA)-to-creatine (NAA/Cr), NAA-to-choline (NAA/Cho), choline-to-creatine (Cho/Cr), and choline-to-NAA (Cho/NAA) ratios were determined in both hemispheres and compared to controls. No significant differences in all metabolite ratios between epileptic dogs and the control group could be found. A time-dependent decrease in the NAA/Cho ratio as well as an increase in the Cho/NAA ratio was found with proximity in time to the last seizure. We found no correlation between metabolite ratios and age or sex in this animal group. Time span from the last seizure to the acquisition of MRS significantly correlated with NAA/Cho and Cho/NAA ratio. We conclude that without a time relation, metabolite ratios in dogs with IE do not differ from those of the control group.

## Introduction

Epilepsy is the most common neurological disease in dogs (1–6). An increasing number of gene defects have been discovered in canine seizure disorders (6, 7), in which abnormal electrical activity arises from structurally intact neural tissue or a microstructurally deviant group of cells, which cannot be visualized in conventional MRI (8). Generally, the diagnosis of canine idiopathic epilepsy (IE) is made without evidence of MRI structural or morphological lesions or insults that secondarily influence the neuronal network (9). This concept might change in the face of growing evidence of documented structural changes in dogs with IE, like changes of apparent diffusion coefficient (ADC) parameters (10) or volumetric changes of white-to-gray matter ratio (11) or hippocampus (12, 13). The main knowledge about generalized tonic–clonic seizures (GTCSs) is based on electrophysiological experiments that indicated involvement of bilateral cortical, subcortical, and brain stem networks in the seizure activity (14–16). Seizures can arise from temporal lobe structures, including the hippocampus and amygdala, representing the most common form of epilepsy in humans and possibly in animals (1, 17–21). In human patients, the associated pathological substrate is usually a disorganization in the temporal lobes, amygdala, and hippocampal cytoarchitecture and gliotic atrophy of the pyramidal cell band of the cornu ammonis fields (hippocampal sclerosis) (22, 23). Alterations of hippocampal neurons and limbic structures have been found in histopathological examinations of the brain in epileptic dogs but were rather the consequence of seizure activity (24–26). Although the role of the hippocampus as a seizure generator in dogs is controversial (27), a growing body of evidence suggests at least some role of the temporal lobe structures in canine epilepsy (2, 11, 28, 29). T2-weighted (T2-w) hyperintense signals and atrophy of the hippocampus could be demonstrated in MRI investigations of epileptic dogs (2, 11, 28, 29), which is an indicator of structural changes of the hippocampus in humans (30, 31). An increased ADC has been measured in the temporal lobe of epileptic dogs (10). Finally, abnormal activity on electroencephalography (EEG), localized within the temporal lobe in dogs with epileptic seizures, has been documented in association with hippocampal atrophy (12, 32). These results warrant further investigation of the temporal region in canines with suspected idiopathic generalized epilepsy, helping to understand the changes of this brain area under seizure exposition.

Proton magnetic resonance spectroscopy (H1-MRS) provides biochemical information about specific brain regions of interest and visualizes alterations in metabolite concentrations before structural changes may be observed. In humans with temporal lobe epilepsy (TLE), it is sensitive in detecting metabolic alterations in dysfunctional epileptogenic regions such as the hippocampal formation before increased T2-signal intensity, loss of volume, and contrast enhancement have set in (33, 34). H1-MRS is widely used in human medicine for the detection of metabolic disturbances indicating neuronal injury/loss in the hippocampus in patients with generalized epilepsy (35–38). H1-MRS has now emerged as a technology with possible benefit for the study of canine brain function (39, 40) and various disease states (41–44) and can potentially provide insight into the cellular and metabolic pathophysiology of the temporal lobe.

The aim of our study was to evaluate the interictal metabolic activity of the temporal lobe region in dogs with IE using H1-MRS and compare the data to those of healthy controls. We hypothesized that dogs with IE would have relevant changes in examined metabolite ratios [N-acetylaspartate (NAA)-to-creatine (NAA/Cr), choline-to-creatine (Cho/Cr), NAA-to-choline (NAA/Cho), choline-to-NAA (Cho/NAA)] in comparison to the healthy group.

## Materials and Methods

### Ethics Statement

The study was conducted according to the University in Giessen (Germany) and in Wroclaw (Poland) institutional guidelines. This study was approved by the institutional animal care and use committee and adheres to the principles for the humane treatment of animals as stated by the Polish Institutes of Health guidelines (45). The owners of the dogs in the study and control group gave their written consent to the enrollment in this study.

### Study Population

The prospective study was performed in the Department of Internal Medicine with a Clinic for Horses, Dogs and Cats, Faculty of Veterinary Medicine, Wroclaw University of Environmental and Life Sciences, Wroclaw, Poland. All dogs underwent clinical and neurological examinations by a veterinary neurologist. Pre-anesthetic laboratory investigations comprised complete blood cell count and serum biochemistry panel, electrolytes as well as fasted ammonia, bile acids, and urinalysis. MRI of the brain was performed in all dogs. The cerebrospinal fluid (CSF) was obtained by puncture of the cerebellomedullary cistern under general anesthesia immediately after the MRI examination. Cytologic and biochemical analyses of the CSF including specific gravity, electrolytes, leukocyte count, complete blood cell count, protein concentration, Pandy's reaction, and glucose concentration were performed. Except bile acids, ammonia testing, all investigations were also performed in the control group.

Only dogs diagnosed with IE that manifested generalized tonic–clonic seizures were included in the study group. The diagnosis of IE was based on: (1) the clinical history of repeated seizures based on the anamnesis with the owner and the presence of a seizure diary for evaluation in dogs, with a special focus on the last observed generalized seizure occurrence; (2) the age between 6 months and 6 years at the time of the first seizure onset; (3) unremarkable interictal physical and neurological examinations; (4) normal complete blood cell count, biochemistry panel, and electrolytes, as well as fasted serum ammonia and bile acids, CSF examination, and urinalysis; (5) normal MRI of the brain, performed according to the International Veterinary Epilepsy Task Force recommendations (46). All patients included in the study group did not receive any epileptic treatment at the time of the examination.

Cases suspected of paroxysmal events other than epileptic origin (e.g., cardiogenic syncope, vestibular disease, narcolepsy) were excluded. Dogs included in the control group underwent the examination of the cervical spine for various clinical reasons and were included in the study if having no history of epileptic seizures and fulfilled the abovementioned inclusion criteria.

### Anesthetic Protocol

Each patient underwent premedication using medetomidine [0.02–0.03 mg/kg intramuscularly (i.m.)] with butorphanol (0.1–0.4 mg/kg). General anesthesia was induced with propofol [4–8 mg/kg intravenously (i.v.)] and maintained by inhaled anesthetics (1.8% isoflurane in oxygen).

### Magnetic Resonance Imaging

All MRI and MRS data were acquired in ventral recumbency with a 1.5-Tesla MR scanner (Ingenia; Philips Medical Systems, 2013) using Stream HeadSpine 15-channel coil (Philips Medical Systems, 2013). All animals enrolled in the study underwent a standardized MRI protocol (International Veterinary Epilepsy Task Force-IVETF recommended protocol) that included 2-mm T2-w spin echo (SE) images, 2-mm fluid-attenuated inversion recovery (FLAIR) sequence, T2^*^ sequence, 0.8-mm T1-weighted three-dimensional (3D), pre- and post-contrast images, following intravenous administration of gadolinium (Gadobutrol 604.72 mg/mmol, solution for injection). All MR sequences were performed as follows: conventional transverse and sagittal T2-w images [repetition time (TR) 4,423.5 ms/echo time (TE) 100 ms], dorsal FLAIR (TR 9,000 ms/TE 140 ms/inversion time (TI) 2,450 ms), 3D T1-w pre- and post-contrast images (TR 25 ms/TE 5.1 ms).

### Magnetic Resonance Spectroscopy

**Figure 1:**
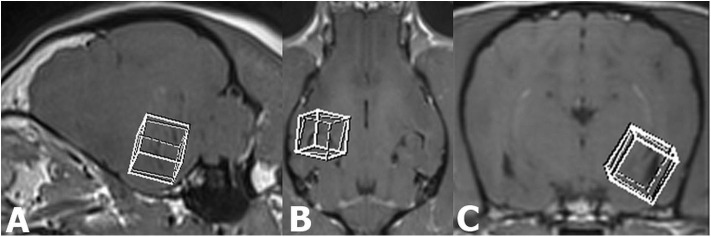
Sagittal **(A)**, dorsal **(B)**, and transverse (**C**) T1-weighted MR images illustrating the voxel placement in the temporal lobe including the hippocampus and part of the amygdala.

In all dogs, an additional single-voxel proton MRS was introduced to the standard epileptic MRI protocol performed before intravenous administration of the contrast medium. The MRS studies were targeted to regions of interest (temporal lobe, hippocampal area; Figures 1A–C). A cubic voxel (10 ×10 ×10 mm) was identical for all studies and manually positioned bilaterally, symmetrically at a thin, 0.8-mm T1-w 3D pre-contrast dorsal, sagittal, and transverse into the region of left and right temporal lobe, including a middle part of the hippocampus and the amygdala.

Voxel positioning was carefully done, with avoidance of the temporal bone, and lateral ventricle inclusion to prevent unwanted signal contributions or chemical shift contamination from non-nervous tissues. Volume selective excitation [point resolved spectroscopy (PRESS)] with chemical shift-selective (CHESS) pulse for water suppression (bandwidth 50 Hz) was used. The MRS studies were acquired as spin echoes with a TR of 2,000 ms, a TE of 144 ms, and a number of phase-encoding steps (NP) of 1.024. A total of 256 images were averaged for an acquisition time of 10 min for each investigated side of the mesial temporal/middle hippocampal/amygdala area.

### Spectrum Analysis

**Figure 2:**
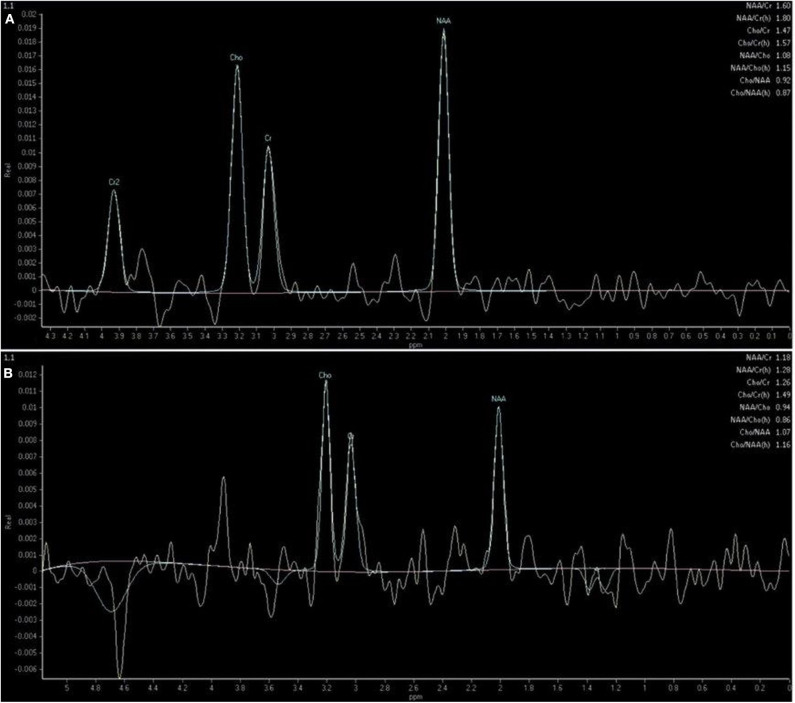
Single-voxel spectrum acquired at the level of the temporal lobe showing metabolite peaks of N-acetylaspartate (NAA) at 2.0 ppm, creatine (Cr) at 3.0 ppm, choline (Cho) at 3.2 ppm and the calculated metabolite ratios in a mixed-breed dog with degenerative disc disease included in the control group **(A)** and in a 5-year-old mixed-breed male dog with idiopathic epilepsy and last seizure 7 days ago **(B)**.

MRI studies were anonymized and presented in a randomized order. Metabolite peaks were identified according to their resonance frequency position along the horizontal axis. The analyzed metabolites included NAA, Cho, and Cr (Figures 2A,B), as they were the only repeatable and readable metabolites detected *via* 1.5-Tesla scanner.

An automatic and operator-non-dependent data processing system was used to obtain the spectra recorded for our study (SpectroView Analysis software, Philips Healthcare). Spectral post-processing included baseline adjustment, noise level filtering, metabolite peak calibration, and the application of spectral plotting, followed by Fourier transformation. The desired signals of metabolite peaks were detected as follows: NAA at 2.02 ppm, Cho at 3.22 ppm, and Cr at 3.02 ppm. The relative ratios of NAA/Cr, Cho/Cr, NAA/Cho, and Cho/NAA were automatically calculated for both groups using resonance areas.

### Statistical Analysis

The collected data were analyzed using statistical software (BMDP/Dynamic, Statistical Software, Inc., Release 8.1). Descriptive statistics were calculated for peak area ratios at MRS. The statistical evaluation included the four metabolite ratios (NAA/Cho, NAA/Cr, Cho/Cr, and Cho/NAA ratios), evaluated equally for both groups. Normal distribution of the data was assessed using a Kolmogorov–Smirnov test. The mean and standard deviation (SD) for the metabolite ratios (NAA/Cho, NAA/Cr, Cho/Cr, and Cho/NAA ratios) for the left and right side were calculated. A two-factor repeated-measures ANOVA test with respect to a side was used to compare the ratio values of the control to those of the epileptic dogs group. A three-factor analysis of covariance (ANCOVA) with repeated measure with respect to a side was used to examine the correlation between the metabolite ratios and the covariates age and sex in the groups. A single-factor ANCOVA with repeated measures with respect to a side of the voxel was applied to examine a possible correlation between the values of the metabolite ratios, seizure duration before examination, and time from the last seizure. The level of significance (α) was *P* <0.05 for all applied tests.

## Results

The IE group comprised in total 27 dogs of different breeds with diagnosed IE and structurally normal MRI of the brain. A list of breeds, age, and gender in the group is given in Table 1. Fifteen dogs in the IE group were male, and 12 were female. Ten dogs without brain disease were included in the control group (Table 2). The mean age of the IE dogs was 3.5 years (with a range from 1 to 7 years), whereas the mean age of the control group was 2.7 years (with a range from 1 to 4 years). All dogs in the IE group showed GTCS with symmetrical bilateral involvement, salivation, and urination.

**Table 1:** Number of different breed dogs comprised in the idiopathic epilepsy group.

**Breed**	**Sex**	**Age in years**	**Days from the last generalized seizure**	**Duration of epilepsy in days**
Siberian husky	F	2.50	28	42
Rottweiler	M	2.00	28	152
Central Asian shepherd dog	F	2.00	27	62
Bavarian mountain dog	M	4.00	21	30
Border collie	F	1.00	21	90
Berger Blanc Suisse	M	2.00	20	30
Beagle	M	2.00	20	360
Dogo Argentino	M	1.50	15	165
Hungarian vizsla	F	3.00	15	130
Mixed-breed dog	F	7.00	15	95
Mixed-breed dog	F	5.00	14	80
Cocker Spaniel	F	4.00	14	195
Golden retriever	M	7.00	14	140
Mixed-breed dog	F	7.00	14	180
French bulldog	M	3.00	12	60
French bulldog	F	3.00	9	92
Mixed-breed dog	M	5.00	7	120
Miniature pinscher	F	1.60	7	29
American Staffordshire terrier	M	2.00	7	210
Staffordshire bullterrier	M	2.00	7	360
Border collie	F	7.00	4	31
Berger Blanc Suisse	M	2.50	2	90
Bullterrier	M	5.00	1	21
Bullterrier	M	2.00	1	365
Labrador retriever	M	1.50	1	160
Labrador retriever	F	3.00	0	7
American Staffordshire terrier	M	7.00	0	336

*M, male; F, female*.

**Table 2:** Dogs included in the control group.

**Breed**	**Sex**	**Age in**	**Diagnosis**
		**years**	
Labrador retriever	M	2.50	Fibrocartilaginous embolism
West Highland white terrier	M	2.40	Degenerative disc disease
Mixed-breed dog	M	4.00	Degenerative disc disease
West Highland white terrier	F	1.00	Spinal trauma
Labrador retriever	F	2.00	Lumbosacral stenosis
Labrador retriever	F	2.50	Fibrocartilaginous embolism
Beagle	F	3.00	Knee trauma
Border collie	F	3.00	Lumbosacral stenosis
Mixed-breed dog	F	3.00	Degenerative disc disease
Maltese	F	3.50	Degenerative disc disease

*M, male; F, female*.

No significant differences were observed when comparing the metabolite ratios between the control and IE group (Table 3). Moreover, the ratios for the left and right regions of interest similarly did not vary significantly between the examined groups. There was no significant influence of age for all examined metabolite ratios (NAA/Cr: *P* = 0.598; Cho/Cr: *P* = 0.862; NAA/Cho: *P* = 0.898; Cho/NAA: *P* = 0.788). Similarly, no significant correlation between sex and metabolite ratios was found (NAA/Cr: *P* = 0.0633; Cho/Cr: *P* = 0.517; NAA/Cho: *P* = 0.065; Cho/NAA: *P* = 0.063).

**Table 3:** Mean, standard deviation, and *P*-values for calculated brain metabolite ratios [N-acetylaspartate (NAA)/creatine (Cr), choline (Cho)/Cr, NAA/Cho, Cho/NAA] of dogs included in the idiopathic epilepsy and control groups.

	**NAA/Cr left**	**NAA/Cr right**	**Cho/Cr left**	**Cho/Cr right**	**NAA/Cho left**	**NAA/Cho right**	**Cho/NAA left**	**Cho/NAA right**
**Idiopathic epilepsy group**	1.31 ± 0.18	1.4 ± 0.24	1.26 ± 0.18	1.29 ± 0.13	1.06 ± 0.21	1.12 ± 0.26	0.97 ± 0.18	0.93 ± 0.2
Difference right–left hemisphere	*P* = 0.072		*P* = 0.794		*P* = 0.149		*P* = 0.103	
**Control group**	1.4 ± 0.27	1.48 ± 0.23	1.26 ± 0.21	1.24 ± 0.28	1.13 ± 0.18	1.2 ± 0.19	0.91 ± 0.16	0.85 ± 0.14
Difference left–right hemisphere control	*P* = 0.122		*P* = 0.264		*P* = 0.121		*P* = 0.128	
Difference idiopathic epilepsy vs. control group	*P* = 0.228		*P* = 0.703		*P* = 0.314		*P* = 0.251	

A significant positive correlation was found between NAA/Cho ratio (*P* = 0.03) and Cho/NAA ratio (*P* = 0.01) and time from last seizure. The regression coefficient was −0.005 in NAA/Cho, indicating higher values of this ratio with closer distance from the last seizure (Table 4). For the Cho/NAA ratio, the regression coefficient was positive (0.008), indicating lower values in dogs with recent seizures. The ratios NAA/Cr (*P* = 0.21) and Cho/Cr (*P* = 0.169) showed no significant correlation with time from the last seizure event.

**Table 4:** Significant positive correlation found between N-acetylaspartate (NAA)/choline (Cho) ratio (*P* = 0.03) and Cho/NAA ratio (*P* = 0.01) and time span from last seizure and the acquisition of magnetic resonance spectroscopy (MRS) in comparison to the control group with relation to the side of the voxel. Calculated regression coefficients show how the metabolite ratio increases [NAA/creatine (Cr), NAA/Cho] and decreases (Cho/Cr, Cho/NAA) over time.

	**Main effects**		
**Metabolite**	**Last seizure**	**Side of the voxel**	**Regression**
**ratio**	**in days**	**(left/right)**	**coefficients**
	***P*-value**	***P*-value**	
NAA/Cr	0.121	**0.054**	−0.005
Cho/Cr	0.169	0.364	0.006
NAA/Cho	**0.026**	0.275	−0.008
Cho/NAA	**0.009**	0.243	0.008

*Significant differences are highlighted in bold*.

## Discussion

In this study, we present data of interictal single-voxel H1-MRS of the temporal lobe with hippocampus and amygdala region in dogs with IE and a structurally normal brain. No significant changes in the investigated metabolite ratios were observed in the IE group compared to the controls if a time span from the last seizure was not taken under consideration. Several studies in human medicine on the degree of metabolite ratio changes in patients during ictal, postictal, and interictal phases were performed (47–49). The common findings include a significant decrease in NAA/Cr and NAA/Cho ratios in the affected brain region, usually during ictal and postictal phases. Metabolite disturbances tend to normalize in the affected area in the postictal and interictal period, strongly suggesting rather the functional changes of neurons (48, 49). However, in one study, no significant differences in metabolite ratios in the postictal and interictal phases were observed (47). It is possible that examined metabolite ratios normalized in the interictal phase or there were primarily no metabolite ratio abnormalities in the examined region of interest.

A relation between metabolite ratios and the time from the last seizure was found, i.e., the closer the MRS of the temporal lobe was obtained from the time of the last seizure, the lower was the Cho/NAA ratio. NAA/Cho ratio was analogously higher in dogs with recent seizures and decreased over time. The significantly higher NAA/Cho ratio in our study in dogs with recent seizures is potentially caused by higher/stable NAA concentration or lower choline concentration in the examined brain region.

The major brain metabolites analyzed with long echo time H1-MRS of brain tissue using 1.5-Tesla MRI include NAA, Cho, and Cr, as these metabolites have the most prominent and readily identifiable peaks. NAA, Cho, and Cr compounds could represent the neuronal, astrocytic, and mitochondrial associated dysfunction in epilepsy and therefore were selected for further investigation in our patients (50–52). NAA is synthetized from l-aspartate and acetyl coenzyme A mostly in neurons (53, 54). Various NAA-fixation staining techniques allowed the visualization of NAA at different levels with different neuronal populations including higher levels in pyramidal neurons with longer axons and more extensive myelination than in smaller interneurons (55, 56). NAA is one of the most abundant amino acids in the central nervous system and is involved in energy metabolism (57). It has also been suggested to play a role in the synthesis of myelin and lipids in the central nervous system (58). Loss of NAA signals is considered to be consistent with loss of neuronal integrity but can also be used as a marker of impaired neuronal function and reduced oxidative metabolism (59). Increase in NAA concentration as a result of toxic buildup of NAA and secondary cell death or impaired brain metabolism associated with the inability to catabolize NAA can be related to osmotic stress (60, 61). Significantly higher NAA/Cho ratio in the presented study could potentially be caused by higher/stable NAA in canines with recent seizures. High concentrations of NAA delivered to the lateral ventricle of rats were found to induce seizures (62, 63). Further MRS studies during the ictal period are needed to evaluate these changes. Cho is an essential metabolite responsible for the synthesis of cell membranes and its degeneration (membrane turnover). Moreover, Cho is a precursor of phosphocholine, and acetylcholine and is involved in lipid transport and methyl-group metabolism (64). The concentration of Cho represents cellular density and cell water turnover, and it differs depending on the localization. Excessive concentrations of acetylcholine and its precursor Cho were also found in the cortex and hippocampus in rat brain during epileptic seizures and were responsive to pharmacological manipulation (65). The decreased level of Cho results in lower acetylcholine synthesis and decreased concentration in the hippocampus leading to impaired sleep and memory (66). A significant change in NAA/Cho ratio in our study on dogs with recent seizures could also suggest a secondary lower level of Cho and acetylcholine in our dogs. Cr plays an essential role in energy synthesis being converted by creatine kinase (CK) to phosphocreatine (PCr), which is directly used in a production of adenosine triphosphate (ATP) (61, 67). Cr is synthesized in axonal mitochondria, oligodendrocytes, dendrites, synapses, and neuronal cell bodies. Total Cr is often applied as an internal reference for MRS due to its relative stability in both normal and pathological brain tissue (68–71). Cr spectrum signal was also found significantly reduced in MRS in animal models of epilepsy (72) and in a canine model of pentylentetrazole-induced generalized seizures (73).

In human patients with IE, spectroscopic studies of the posterior hippocampus, amygdala, and mesial temporal lobe area are mainly used to determine the lateralization of the epileptogenic zone. The unilateral decrease in NAA/Cr, NAA/Cho, or Cho/Cr ratios showed good concordance with the localization of the epileptogenic zone in the respective hippocampus (35, 36) and predicts a positive surgical outcome (74). Our study showed no significant changes in metabolite ratios between the left and right regions of interest. Furthermore, resection of the hippocampus is also performed in patients with structural or functional extra-temporal seizure foci if changes in the hippocampus can be detected (75–77). The presence of hippocampal damage and/or dysfunction, in association with a lesion outside the temporal lobe, is assumed to be due to the propagation of epileptogenic activity from the remote focus into the hippocampus formation, inducing secondary epileptogenic effects (78). Interictal spectroscopic examination revealed reduced hippocampal NAA/Cr and NAA/Cho in humans with extra-temporal epilepsy (37, 78).

Our understanding of epilepsy in dogs is still limited and somewhat focused on the existence of primary TLE (8). However, the proof of any negative effect of the hippocampus on seizure generation or enhancement in epileptic dogs might take us a step forward and encourage veterinary neurosurgeons to consider resection of the temporal lobe in dogs with refractory epilepsy (13). The finding of this study, namely, that GTCS did not create long-term changes in metabolic spectra measured in the mesial temporal region including middle hippocampus, may suggest that repeated seizures do not cause secondary damage in this area in dogs. Alternatively, changes were observed only in the short time period after the seizure (79). Another possibility is that dogs that did not show changes in the MRS of this area were suffering from an epileptogenic zone in a different brain region. Extensive studies on GTCS in human medicine presented thalamo-cortical dysfunction with the GTCS-associated necrosis in thalamic relay neurons (80) as well as abnormal functional connectivity between various cortical and subcortical regions including cingulate cortex and temporal and olfactory lobes (16). The MRS studies of thalamus in patients with absence epilepsy showed decreased NAA/Cr ratio, suggesting neuronal injury in this area (81). The focus of this research did not involve the investigation of adjacent areas or other regions of the brain. Placement of the voxel in the temporal lobe area in dogs with GTCS in the study group was based on recent studies in veterinary medicine, which suggested the active involvement of those structures in the development of GTCS. Metabolic alterations in the thalamus, cingulate cortex, as well as other cortical areas should be further studied in canines with IE to examine the origin of GTCS and compare those results.

However, the fact that other studies documented functional or structural changes of the hippocampus (10, 12, 13, 28) warrants further investigations limited to hippocampus formation using H1-MRS. Further detailed classification of dogs with focal seizures and focal seizures evolving to generalized seizures, as well as use of continuous long-term electroencephalography, should be considered in order to improve anatomical localization of epileptic activity.

The results of our study might have been limited due to a number of reasons. Although we could not document a correlation between the duration of seizure activity before imaging, the influence of seizure frequency and duration cannot be ruled out. Humans with TLE, studied within 24 h postictal period, showed no changes in metabolite ratios, suggesting that these ratios are insensitive to immediate seizure history (47, 82). Another study found no relationship between NAA/Cho, NAA/Cr, and seizure duration, frequency, or lifetime estimated seizures (39). We found a correlation between time distance to the last seizure and decrease in NAA/Cho. If all dogs would have been examined at a comparable time distance to the last seizure, the differences in the metabolite ratios might have become significant. The difference between ratios between left and right hippocampus formation almost reached significance, which might also increase with standardized imaging modalities. These possible connections should be further investigated. Metabolite ratios can also differ along the long axis of the hippocampus with lower ratios of NAA/Cho and NAA/Cr in the anterior as compared with the posterior part of the hippocampus (83).

We decided for single-voxel measurement as it provides volume selectivity of the acquired signal and therefore high signal-to-noise ratio, resulting in high-quality spectra suitable for quantitative analysis. However, using single-voxel acquisition, it was technically not possible to reduce the voxel <1 cm in size. Therefore, the spectra were not measured exclusively in the temporal lobe but contained information from adjacent brain tissue, especially from the thalamus. Overlapping the thalamus however, in our opinion, produces much less signal contamination in comparison to overlapping the calvaria and producing poor-quality spectra due to fat contamination (84). This can be seen as a systematic error that occurred in every examined dog, but still with the use of 1.5-Tesla magnet and 1-cm voxel placing, it was technically unavoidable and still could be used as a reference for further studies.

Multi-voxel acquisition techniques offer the advantage of examining smaller regions of interest, which is important when studying small or irregularly shaped anatomic structures such as the hippocampus formation. However, spectral contamination from adjacent voxels can have a high influence on the values, which makes quantification of the spectra difficult (54). Ratios determined by Warrington obtained in the temporal lobe of 10 healthy beagles using multi-voxel acquisition were higher than those in our control group (39). Both absolute values and NAA/Cho, NAA/Cr, and Cho/Cr ratios have a larger range and median values, most likely reflecting the differences of single- vs. multi-voxel acquisition. Nevertheless, these MRS results should be compared with caution, given that ratios obtained by Warrington were evaluated using 3-Tesla MRI. Metabolite ratios may vary depending on the type of the software, field strength, and protocol type for MRS (85). On the other hand, in the study by Kim et al. (86), no significant differences were observed between the two field strengths.

Systematic comparison between spectroscopic data obtained by single- and multi-voxel acquisition has been performed in dog cadavers (84). According to the recent preliminary findings (87), single- and multi-voxel spectroscopy techniques yield comparable results for similar sized regions of interest in the normal canine brain.

The absolute metabolite quantification can be obtained in two different ways: as ratios or relative metabolite concentrations (relative to water content of the brain) (54). The results of our spectra are expressed in terms of ratios, which is considered to be a stable indicator to describe parenchymal changes in humans (34, 88). Metabolite ratios such as NAA/Cr and Cho/Cr have been used commonly for quantification, instead of relative metabolite levels (71, 83). However, the use of metabolite ratios potentially increases the variability of the result compared with the individual components over half the time (88).

In contrary, relative metabolite concentrations can be measured using the so-called linear combination model (LCModel). LCModel analysis method is particularly attractive for the evaluation of the uncertainty (e.g., Cramer-Rao lower bounds), which estimates the quality of the spectra used for further analysis. The dedicated LCModel software is widely available in human medicine and also has been already used in some veterinary medicine studies (40–44). The main advantage of the LCModel is that the software is highly automated, has built-in corrections, and requires little user input. It can estimate concentrations of even minor metabolites to high internal precision, which enables to detect a broader spectrum of metabolites of interest. The LCModel is used to analyze the complete spectra, rather than individual peaks, which is an advantage that enables to distinguish two metabolites in one frequency region with nearly identical spectra but different signals in other parts of the spectrum (89). To our knowledge. There is no study in veterinary medicine evaluating the use of LCModel in epileptic canines. Future MRS studies on epilepsy in dogs should focus on comparison of both quantification techniques.

Among the single-voxel techniques, PRESS and stimulated echo acquisition mode (STEAM) are generally used for MRS. Molecules selected for our study (NAA, Cho, Cr) can only be detected on 1.5-Tesla field strength at long echo times. Thus, long echo time PRESS sequence was used in our study because of the improved signal intensity-to-noise ratio (SNR) and a more readable spectrum which contains less signal from the lipid and metabolites with short T2 values (54). In contrary, STEAM technique is favorable for exposure of small metabolites with shorter T2 times (glutamine, glutamate, myoinositol) (90, 91). Nevertheless, a higher field MR scanner is required for the examination at short echo T2 times. Metabolites with shorter T_2_ relaxation times are not resolved from each other at 1.5 Tesla, thus cannot be observed on the spectrum. Glutamate, glutamine, and myoinositol as well as lipids could have not been measured in studied dogs. Further investigation is necessary to present if these compounds would be observed in the interictal phase in dogs with seizure activity. Several studies in human medicine presented increased lactate and myoinositol and decreased glutamate and glutamine in patients experiencing active seizures (48, 51). Further studies on epileptic dogs examining metabolites at short and long echo times using PRESS and STEAM techniques simultaneously need to be performed.

It is also unclear as to whether the measured spectra and ratios might be breed-specific or breed specifically biased due to different skull conformations and brain volume. We included two brachycephalic dogs in our IE group. Curved shape of the analyzed region might have caused slight changes in tissue components in the examined voxels, as the hippocampal volume differs significantly in size in allometric relationships to brain and body weight (92), but there are no data on this issue. Comparisons between larger cohorts of homogeneous groups of dogs of the same breed will be necessary to prove such breed influence on MRS spectra.

It was shown that magnetic field strength has a dependent influence on the measured spectra (93). With the increase of magnetic field strength, an increase in absolute chemical shift is also induced so that the metabolite peaks can be better delineated. When the magnetic field strength is doubled from 1.5 to 3 Tesla, the signal of the metabolite peaks also increases, since the SNR depends on the magnetic strength (85). Future veterinary studies comparing diagnostic accuracy of the obtained spectra at 3 Tesla with 1.5 Tesla should be considered; however, 1.5-Tesla magnets are currently the most available for veterinary patients.

Interictal single-voxel proton MRS of the mesial temporal lobe, middle hippocampus, and amygdala area in dogs with IE and structurally normal MRI revealed no statistically relevant changes in examined metabolite ratios (NAA/Cr, Cho/Cr, NAA/Cho, Cho/NAA) in comparison to the healthy group. However, the NAA/Cho and Cho/NAA ratios were correlated with time duration to seizure. Our findings support the concept that MRS provides further information about the status of brain function and proves reversible metabolic dysfunction.

## Data Availability Statement

The datasets used and analyzed during the current study are available from the corresponding author on reasonable request.

## Ethics Statement

The animal study was reviewed and approved by Local Ethical Committee of the Justus Liebig University Gießen, Germany and National Ethics Committee on Animal Experimentation, Poland. Written informed consent was obtained from the owners for the participation of their animals in this study.

## Author Contributions

AO participated in the design of the study, acquisition of data, and drafting of the manuscript. MW, PP, and JN made a substantial contribution to the acquisition of data. KF performed the statistical analysis. MW and MS: made a substantial contribution to the design of the study and helped to draft the manuscript. All authors read and approved the final manuscript.

### Conflict of Interest

The authors declare that the research was conducted in the absence of any commercial or financial relationships that could be construed as a potential conflict of interest. The reviewer IC declared a past co-authorship with one of the authors MS to the handling editor.

## Abbreviations

ADC: apparent diffusion coefficient
ANCOVA: analysis of covariance
ANOVA: analysis of variance
ATP: adenosine triphosphate
CHESS: chemical shift-selective
Cho: choline
Cho/Cr: choline-to-creatine ratio
Cho/NAA: choline-to-N-acetylaspartate ratio
CK: creatine kinase
Cr: creatine
CSF: cerebrospinal fluid
EEG: electroencephalography
FLAIR: fluid-attenuated inversion recovery
GTCS: generalized tonic–clonic seizure
H1-MRS: proton magnetic resonance spectroscopy
IE: idiopathic epilepsy
IVETF: International Veterinary Epilepsy Task Force
LCModel: linear combination model
MRI: magnetic resonance imaging
MRS: magnetic resonance spectroscopy
NAA: N-acetylaspartate
NAA/Cho: N-acetylaspartate-to-choline ratio
NAA/Cr: N-acetylaspartate-to-creatine ratio
NP: number of phase-encoding steps
PCr: phosphocreatine
PRESS: point resolved spectroscopy sequence
SD: standard deviation
SE: spin echo
SNR: signal intensity-to-noise ratio
STEAM: stimulated echo acquisition mode
T1-w: T1-weighted
T2-w: T2-weighted
TE: echo time
TI: inversion time
TLE: temporal lobe epilepsy
TR: repetition time.
